# Cytotoxic Profiles of Beauvericin, Citrinin, Moniliformin, and Patulin and Their Binary Combinations: A Literature-Based Comparison and Experimental Validation in SH-SY5Y Cells

**DOI:** 10.3390/toxins17030143

**Published:** 2025-03-17

**Authors:** Claudia Moyano-López, Luna Bridgeman, Cristina Juan, Ana Juan-García

**Affiliations:** Laboratory of Food Chemistry and Toxicology, Faculty of Pharmacy and Food Science, University of Valencia, Av. Vicent Andrés Estellés s/n, 46100 Burjassot, València, Spain; claudia.moyano@uv.es (C.M.-L.); luna.bridgeman@uv.es (L.B.); ana.juan@uv.es (A.J.-G.)

**Keywords:** mycotoxin, SH-SY5Y cell line, cytotoxicity, in vitro, mycotoxin mixture

## Abstract

Mycotoxins are toxic compounds found in food and feed that pose significant risks to human and animal health. This work reviews recent studies on the cytotoxic effects of four mycotoxins: beauvericin (BEA), citrinin (CTN), moniliformin (MON), and patulin (PAT) in various cell lines. Additionally, an experimental study evaluates the effects of these mycotoxins and their binary combinations on human neuroblastoma cells (SH-SY5Y) after 24 and 48 h of exposure using the 3-(4,5-Dimethylthiazol-2-yl)-2,5-Diphenyltetrazolium Bromide (MTT) assay. This analysis is driven by the additional risks posed by the frequent occurrence of these combinations in agricultural and food products, as well as the lack of studies addressing their effects, interactions, and regulatory frameworks. This research focuses on comparing the cytotoxicity data obtained in the SH-SY5Y cell line with previously reported findings in the literature for other cell lines exposed to BEA, CTN, MON, and PAT, individually and in binary combination. The literature highlights significant scientific interest in understanding the cytotoxic effects of these mycotoxins, with findings varying based on exposure time and concentration. Experimentally, PAT demonstrated the highest toxicity in SH-SY5Y cells, while MON was the least toxic. Among combinations, BEA + MON and CTN + PAT showed the greatest reduction in cell viability. However, medium inhibitory concentration (IC_50_) values were not reached for most combinations involving MON, reflecting its lower potency under the studied conditions. These findings underscore the importance of further investigation and enhanced regulations to address the health risks posed by mycotoxins, as their cytotoxic effects remain a pressing issue in food safety.

## 1. Introduction

Mycotoxins are natural toxins derived from the secondary metabolism of different species of filamentous fungi, including *Penicillium*, *Aspergillus*, *Fusarium*, and *Alternaria* [1]. These metabolites have grown in importance within the context of food safety and human health due to their harmful effects and presence in agricultural and food products [2]. They stand out for being highly resistant, so they can be formed during the cultivation, harvesting, transporting, and storage of the food from which they come, entering the food chain and representing a major risk in this area [1].

The species of *Fusarium* are producers of beauvericin (BEA) and moniliformin (MON), and both have been found in corn, wheat, rice, barley, and cereals [3,4]. At the same time, the species of *Aspergillus*, *Monascus*, and *Penicillium,* which can be found in cereals, vegetables, spices, nuts, and oilseeds, are producers of citrinin (CTN) [5,6]. Moreover, the species of *Aspergillus, Byssochlamys*, and *Penicillium* can produce patulin (PAT) and can be encountered in products derived from apples [7]. The variability of producer species and the huge variety of different foods they can contaminate are important aspects in studying the risk assessment and the toxicological effects of the combinations’ presence. Given that mycotoxins rarely occur in isolation in food products, there is substantial evidence supporting potential synergistic, additive, or antagonistic effects. Therefore, it is essential to assess their interactions and combined toxicological impact [8,9,10,11]. BEA and MON are produced by *Fusarium* fungi; both are capable of inducing oxidative stress and affecting cell integrity. Therefore, their joint presence in fruit- and cereal-derived products suggests that they could enhance their cytotoxic and genotoxic effects [10,12,13,14,15,16,17]. BEA and PAT have been reported in common food matrices, and the growth of their producing fungi occurs in subtropical latitudes, which demonstrates that they share a common origin [11]. In another scenario, PAT and CTN are both produced by *Penicillium expansum*, making the study of their co-occurrence effects highly relevant [18]. As seen in real-world food matrices, mycotoxins rarely occur in isolation. Combinations such as BEA + MON in cereals or CTN + PAT in fruit-based products represent realistic exposure scenarios.

Toxicological effects attributed to BEA [19,20,21,22,23], CTN [24,25,26], MON [27], and PAT [28,29,30] have been studied, as reported in the literature (Table 1). BEA is an ionophore compound, which increases the ionic permeability of biological membranes by forming a complex with essential cations (Ca^2+^, Na^+^, K^+^), causing time- and concentration-dependent cytotoxicity. In addition, it increases the production of reactive oxygen species (ROS) and lipid peroxidation (LPO), leading to the production of oxidative stress [4,21]. Many studies have demonstrated the nephrotoxic effect of CTN, and it has been found that this mycotoxin stands out for being neurotoxic, embryocidal, and fetotoxic [26,31]. CTN is significantly related to the dysregulation of some genes related to oxidative stress and apoptosis, so both processes can be related to the neurotoxicity that it causes [32]. The molecular mechanism of action has been conducted through in vitro assays and in vivo models. CTN causes cell cycle arrest and DNA damage, triggers autophagy, and induces mitochondrial damage [33]. As for a possible carcinogenic effect, it is classified by the International Agency for Research on Cancer (IARC) in group 3, since evidence in humans and animals is insufficient. Moreover, MON is characterized by being the cause of chromosomal aberrations and micronucleus formation, but data on genotoxicity and carcinogenicity in vivo are very scarce [3,34]. MON toxicity is primarily associated with alterations in cardiac tissue, inhibiting mitochondrial pyruvate and α-ketoglutarate oxidation. This inhibition may represent the main molecular mechanism underlying its toxicity, contributing to cellular dysfunction and tissue damage [35]. According to IARC, MON belongs to group 2B of this classification [36]. Finally, PAT is shown to be cytotoxic and genotoxic [37,38], and the intake of large amounts of it is capable of inducing neurotoxicity, hepatotoxicity, nephrotoxicity, reproductive toxicity, and severe gastrointestinal disorders [37]. In addition, PAT’s main mechanism of toxicity is due to the inhibition of key biosynthetic enzymes and the induction of oxidative stress [37,39]. This mycotoxin enhances the generation of ROS, leading to damage in critical macromolecules, including proteins, enzymes, and DNA, potentially disrupting cellular functions [40]. Additionally, it is considered that there is no evidence of a carcinogenic effect, so it belongs to group 3 of the IARC classification [36,41]. In some scenarios, the combined exposure may result in additive effects, where the combined toxicity is simply the sum of the individual toxicities of each mycotoxin [32,42,43]. For instance, if BEA and MON each independently cause moderate cytotoxicity, their combined exposure could lead to a proportional increase in toxicity. However, it is also possible that antagonistic interactions could occur, where the presence of one mycotoxin may reduce the toxicity of another. For example, MON, known to have relatively low toxicity by itself, might mitigate the effects of another mycotoxin when combined, potentially leading to reduced overall toxicity compared to individual exposures.

**Figure 1:**
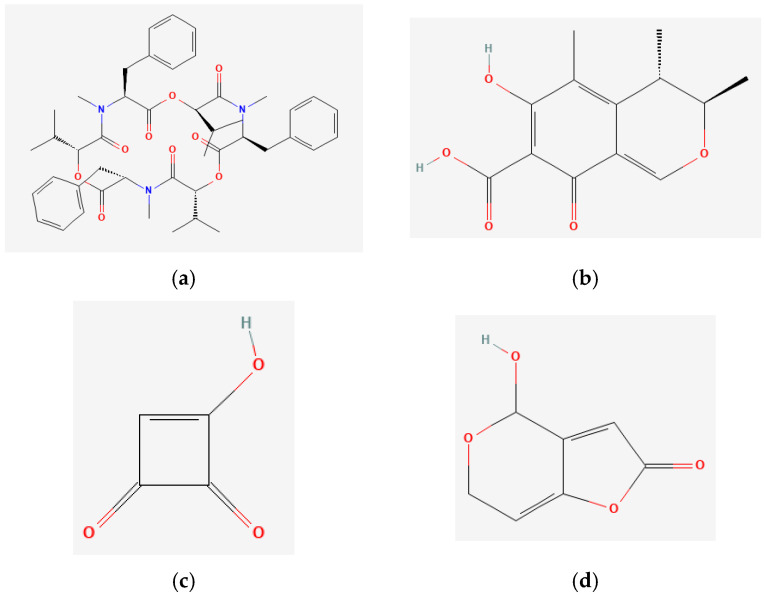
Chemical structures of the mycotoxins (**a**) beauvericin (BEA), (**b**) citrinin (CTN), (**c**) moniliformin (MON), and (**d**) patulin (PAT).

The chemical structures of these mycotoxins are shown in Figure 1.

Since exposure to mycotoxins has been declared as a current problem worldwide, and due to the risks they pose to human and animal health, monitoring and research in this field is of great importance to mitigate food contamination by them [1,44,45,46]. Moreover, the growing interest in mycotoxins and their effects on the nervous system over recent years has drawn attention to chronic exposure and its involvement in diseases such as Alzheimer’s and Parkinson’s [6,26,47]. Although various cell lines, such as human hepatocellular carcinoma cells (HepG2) [48], human embryonic kidney cells (HEK293) [49], Chinese hamster ovary cells (CHO-K1) [11,50], human leukemia T cells (Jurkat T cells) [51], and human fetal intestinal epithelial cells (FHS 74 Int.) [52], have been used in previous studies to evaluate BEA, CTN, MON, and PAT toxicity, there is limited evidence regarding the mechanisms of these mycotoxins’ toxicity in human neuroblastoma cells (SH-SY5Y). The SH-SY5Y cell line is recognized as one of the most widely used in vitro models for research on neurobiological processes, mechanisms of neurotoxicity, and neurodegenerative diseases [53,54,55]. Its human origin, along with its versatility and ease of maintenance, makes it one of the most commonly utilized cell lines in neuronal biology studies. This cell line has the ability to remain in its neuroblast form or differentiate into a neuronal phenotype, which enhances its value in research. This feature allows for the study of toxicity in both developing and fully differentiated cells, making it a flexible and valuable tool for investigating neurotoxic effects at different stages of neuronal maturation [54]. In addition, mycotoxins in their individual form and in their binary combinations are more common in agricultural and food contexts than tertiary or quaternary combinations. Although tertiary (three mycotoxins) and even quaternary (four or more mycotoxins) combinations have been detected, they appear less frequently [9,10]. This is due to numerous factors related to environmental conditions and agricultural practices that facilitate the growth of fungi under similar environmental conditions. Several studies claim that combinations of two mycotoxins are found in 50–70% of the samples tested [9]. Moreover, given the complexity involved in experimenting with tertiary and quaternary combinations, toxicological studies focus on the analysis of the individual and binary toxic effects of mycotoxins in order to try to build a progressive understanding of the toxic mechanisms. Accordingly, the objective of this study is to investigate the exposure of four mycotoxins, BEA, CTN, MON, and PAT, individually and their binary combination in SH-SY5Y cells using the 3-(4,5-dimethylthiazol-2-yl)-2,5-diphenyl tetrazolium bromide (MTT) assay. Furthermore, the results obtained with SH-SY5Y cells are compared with those reported in the literature conducted on different cell lines in order to identify which system is more vulnerable to these mycotoxins both individually and in combination. Recognizing that the study of mycotoxin effects is essential for protecting human and animal health, as well as global food safety, detailed research enables the improvement of detection methods, the establishment of safety regulations, and the design of preventive strategies.

**Table 1:** In vitro cytotoxicity studies on BEA, CTN, MON, and PAT reported in the literature in recent years.

Mycotoxin	Cell Line	Dose and Exposure Time	Results	Ref.
BEA	CHO-K1	0–20 μM 24 h, 48 h, and 72 h	Reduction from 96% to 0% (*p* ≤ 0.05) in cell proliferation in a time-dependent and concentration-dependent manner.	[11]
		0–20 μM 24 h, 48 h, and 72 h	Reduction in cell viability in a time-dependent and concentration-dependent manner. The most significant reduction occurred from 2.5 μM and 0% cell viability is reached (20 μM). IC_50_ (24 h): 10.7 ± 3.7 μM. IC_50_ (48 h): 2.5 ± 3.3 μM. IC_50_ (72 h): 2.2 ± 3.3 μM.	[56]
		1–100 μM 24 h	IC_50_ (24 h): 12.08 ± 1.10 μM.	[50]
	Jurkat T-cells	0–15 μM 24 h, 48 h, and 72 h	Reduction in cell viability in a time-dependent and concentration-dependent manner, reaching values of 50% (after 24 h), 45% (after 48 h), and 25% (after 72 h). IC_50_ (24 h): 7.5 ± 0.4 μM. IC_50_ (48 h): 5.0 ± 0.3 μM. IC_50_ (72 h): 3.0 ± 0.6 μM.	[51]
	RGA cells	0–10 μM 48 h	No cytotoxicity was detected in any of the exposed RGA cell lines, with cell viability consistently maintained between 85% and 100%. However, a significant decrease in cell viability (*p* ≤ 0.001) was observed at a concentration of 10 μM BEA.	[57]
	HepG2	0–25 μM 24 h, 48 h, and 72 h	There was a 1.04-fold increase in cell viability after 48 h and 72 h of exposure to 0.9 μM BEA. This increase was mitigated, most notably, from 8.3 μM (*p* ≤ 0.001). From this concentration, cell viability is less than 50% in all cases.	[58]
	IPEC-J2	0–10 μM 72 h	Cell viability was not significantly reduced for any of the concentrations used. The percentage of cell viability was maintained, in all cases, between 100% and 80%.	[59]
CTN	SH-SY5Y	15–500 μM 24 h	There was a 95% decrease in cell viability with 500 μM (23.27 times lower than the control). IC_50_: 250.90 μM.	[5]
		2-50 μM 72 h	An amount of 50 μM results in a 0.82 relative viability (1.2 times lower than the control). Lower concentrations (2–20 μM) were not significant.	[26]
		0–50 μM 24 h	The higher concentration of CTN used (50 μM) managed to reduce the percentage of cell viability to a value of 70–75%. An amount of 38.75 μM reduced cell viability by 20% compared to control.	[24]
		10–80 μM 72 h	For the maximum concentrations of CTN used, cell viability was considerably reduced, reaching values of 20%.	[32]
	HEK293	20–120 μM 24 h, 48 h, and 72 h	CTN caused a reduction in concentration and was time-dependent. At 80 μM, viability dropped to 54% compared to control (72 h).	[38]
	BoMacs	2.5–320 μM 48 h	IC_50_: 91.20 μM and IC_25_: 52.72 μM.	[60]
	HCT 116	150 μM 24 h	CTN caused a reduction in cell viability (*p* < 0.05), decreasing it to 40%, which is 2.5 times lower than the control.	[61]
	HepG2	0–300 μM 24 h	Decrease in viability depending on the dose (24 h) compared to the control. CTN 50 μM: 25% reduction. CTN 200 μM: 56% reduction. CTN 300 μM: 80% reduction. The study established that the changes detected were significant (*p* < 0.05) in all the concentrations studied, except for CTN 100 μM.	[48]
	FHS 74 Int.	0–20 μg/mL 72 h	CTN did not induce sufficient cytotoxicity and the IC_50_ value could not be determined.	[52]
	TM4 Sertoli	25–200 μM 6 h, 12 h, 24 h, 48 h, and 72 h	After 24 h of exposure, cell viability decreased depending on concentration. The results were significant from 50 μM. From this concentration, the reduction in cell viability occurs in a percentage ranging from 25% to 65% (200 μM) (*p* < 0.001).	[62]
MON	Caco-2	0.2–100 μM 48 h	The maximum concentration tested (100 μM) showed less than 30% inhibition of cell proliferation compared to the control.	[63]
	FHS 74 Int.	0–20 μg/mL 72 h	MON did not induce sufficient cytotoxicity and the IC_50_ value could not be determined.	[52]
	IPEC-J1	0–10 μM 72 h	Exposure to MON did not affect cell viability in any case during the 72 h period.	[59]
	THP-1	0.001–10 μM 48 h	The maximum concentration used (10 μM) of MON showed a 30% reduction in cell viability (*p* < 0.05).	[64]
	HepaRS	0.001–10 μM 48 h	Only a 10% reduction in cell viability was achieved with MON 10 μM (*p* < 0.05).	[65]
PAT	AML-12	4 μM 36 h	PAT 4 μM reduced viability by 25% compared to the control (1.33 times lower cell viability than the control).	[66]
	DBTRG-05MG	10–60 μM 24 h	PAT induced a concentration-dependent decrease, with cell viability being 20 times lower at 60 μM (*p* < 0.05).	[41]
	HEK293	2.5–15 μM 8 h	There was a decrease in cell viability in a dose-dependent manner. Maximum of 63% reduction with PAT 15 μM (*p* < 0.005).	[49]
		−2 μM 24 h, 48 h, and 72 h	PAT showed significant effects from 1 μM (2.2 times lower than the control) after 24 h. PAT 1 μM and 2 μM showed 1.72 and 4.16 times less viability than the control, respectively, after 48 h. After 72 h, these were 2.2 times lower and 10 times lower than the control with concentrations of 1 μM and 2 μM, respectively.	[38]
	V79	0.35–1.55 μM 24 h	Only from 1 μM was there a significant reduction in viability. Regarding control, 0.35 μM PAT showed 1.17 times less viability; 0.95 μM showed 1.21 times less viability; 1.55 μM showed 5 times less viability.	[67]
	CHO-K1	0–6.25 μM 24 h, 48 h, and 72 h	Significant reduction in cell viability at higher exposure concentrations (*p* ≤ 0.05). An amount of 3.125 μM showed 45% cell viability and 6.25 μM showed 16% cell viability.	[11]
		0.2–25 μM 24 h	The IC_50_ values for PAT were 0.69 ± 0.03 μM, respectively. PAT inhibited the growth of CHO-K1 cells more strongly than BEA.	[50]
	BoMacs	0.0038–4.8 μM 48 h	Cytotoxicity was significant at concentrations greater than 2.4 μM for PAT. IC_50_: 0.56 μM and IC_25_: 0.32 μM.	[60]
	HepG2	1–100 μM 48 h	Reduction in metabolic activity in a dose-dependent manner. IC_50_: 8.43 μM. After reaching this value, there was no dose-dependent decrease in metabolic activity and the cells were viable, albeit at low levels.	[37]
		0–30 μM 24 h	Dose-dependent relationship between PAT concentration and cytotoxic effect.	[68]
	NRK52E	0–5000 nM 24 h	With PAT 50 and 100 nM, viability increased by about 20–30% with respect to the control value, since up to 500 nM there was no significant loss of cell viability. These results suggest that a low concentration of PAT may increase cell proliferation; however, higher concentrations (1–5 μM) of PAT significantly caused cytotoxicity (*p* < 0.001).	[69]
BEA + PAT	CHO-K1	BEA: 0.156–1.25 μM PAT: 0.049–0.39 μM BEA + PAT [3.2:1] 24 h, 48 h, and 72 h	The combination is equally cytotoxic when they are tested separately. In addition, at none of the exposure times was cell viability less than 75%.	[11]
CTN + PAT	BoMacs	IC_25_	It did not cause a significant effect on the percentage of cell viability.	[60]

AML-12: mouse hepatocyte cell line, BoMacs: bovine macrophage cell line, Caco-2: human colon adenocarcinoma cells, CHO-K1: Chinese hamster ovary cells, DBTRG-05 MG: human brain multiform glioblastoma cell line, FHS 74 Int.: human fetal intestinal epithelial cells, HEK293: human embryonic kidney cells, HepaRS: human liver cell line, HepG2: human hepatocellular carcinoma cells, HCT 116: human colorectal carcinoma cells, IC25: inhibitory concentration 25, IC_50_: half maximal inhibitory concentration, IPEC-J2: pig small intestine epithelial cells, Jurkat T-cells: human leukemia T cells, NRK52E: rat renal epithelial cells, RGA cell lines: activated genetic reporter cells, SH-SY5Y: human neuroblastoma cell line, THP-1: human monocytic cell line, TM4 Sertoli: Sertoli cells derived from mouse testis, V79 cells: lung fibroblasts from male Chinese hamster.

## 2. Results

### 2.1. State of the Art of Cytotoxicity Assays with BEA, CTN, MON, and PAT

A recent literature review was carried out to assess the cytotoxic impact of the targeted mycotoxins on various cell lines of different origins (comprising the last 24 years).

The most relevant data extracted from the included scientific articles are synthesized in Table 1. The collected information was structured into five aspects: mycotoxin studied, cell line analyzed, exposure dose, exposure time, and results obtained. It was observed that the number of articles about cytotoxicity assays of the four mycotoxins (BEA, CTN, MON, and PAT) was higher for individual studies compared to combined exposure studies. Consequently, it is noticeable that toxicological studies contemplating combined presence are necessary due to current real consumption scenarios including complex food mixtures that present multiple mycotoxin contamination. Current mycotoxin regulation mainly relies on toxicological data from studies focused on their individual exposure without considering the combined exposure to multiple mycotoxins. The toxicity of mycotoxin mixtures cannot always be predicted from individual data, as their interactions may be synergistic, additive, or antagonistic. This research gap leaves the health risks of simultaneous exposure to multiple mycotoxins poorly understood. In response, the European Food Safety Authority (EFSA) is prioritizing the development of risk assessment models associated with the combination of multiple mycotoxins, aiming to generate a better approach to this problem, improving food safety knowledge and understanding and providing better risk management strategies in real-life scenarios involving complex food mixtures [70,71].

Furthermore, the significance of this literature review lies in its highly valuable point of view in in silico approaches to research, since it provides a theoretical and experimental framework that serves as a guide in this field of research. The in vitro studies, reported in Table 1, and the results proposed in our article can contribute to improving the interactive database used to assess chemical hazards by predicting different toxicological endpoints [19].

### 2.2. Cytotoxicity Assay of the Individual Treatment of BEA, CTN, MON, and PAT in the SH-SY5Y Cell Line

**Figure 2:**
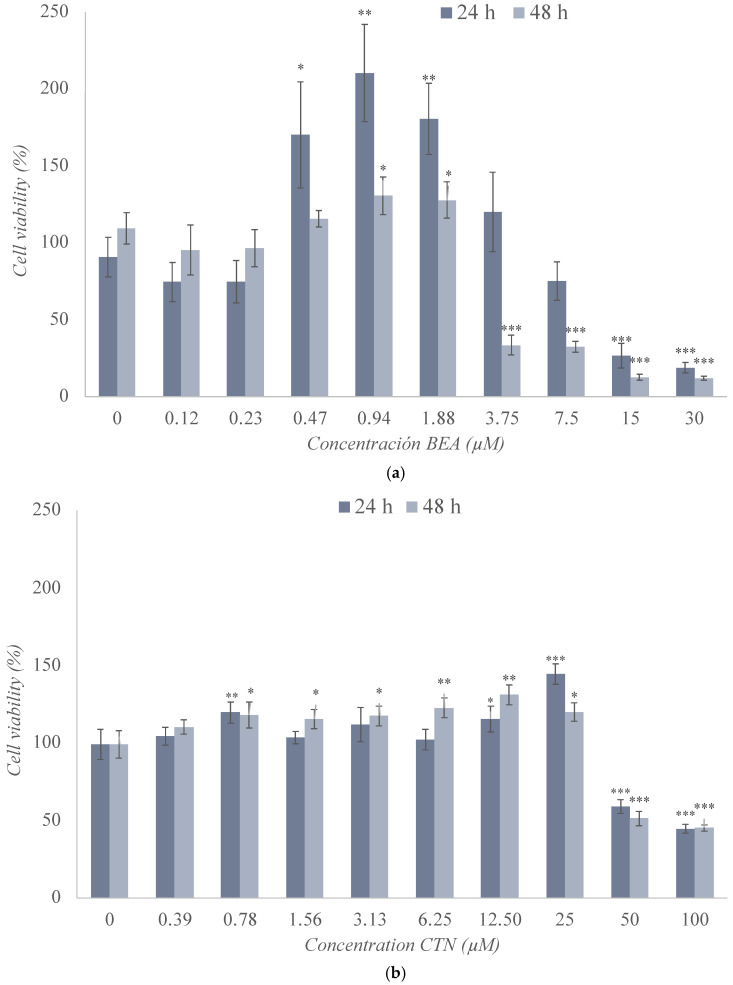

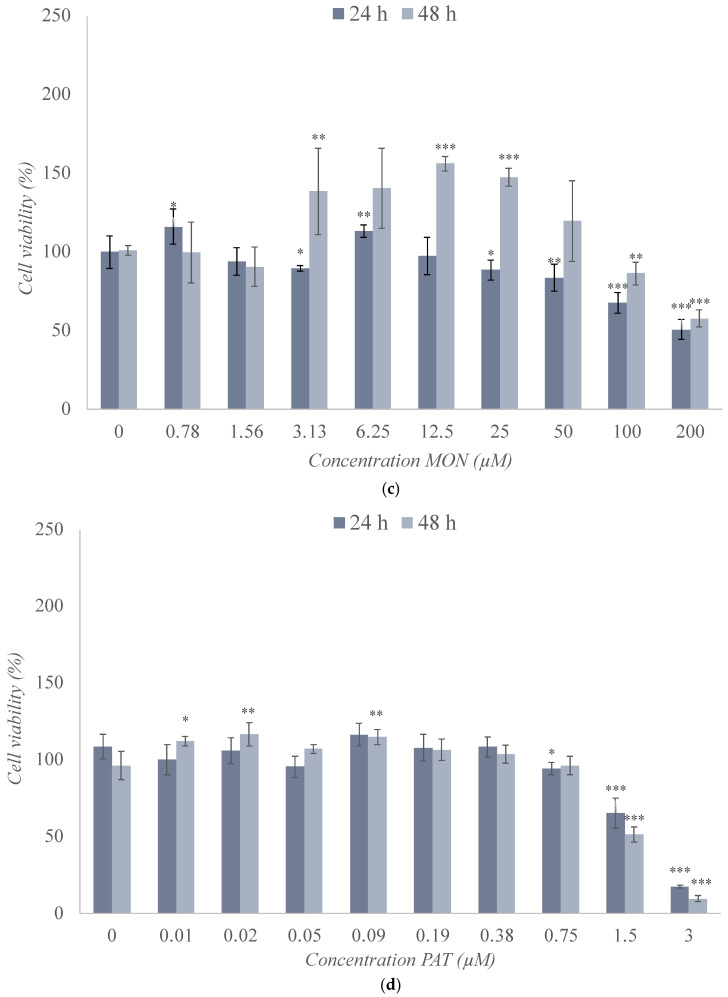
Cytotoxic effect of (**a**) beauvericin (BEA), (**b**) citrinin (CTN), (**c**) moniliformin (MON), (**d**) and patulin (PAT) in SH-SY5Y monolayer cultures obtained by MTT assay after 24 h and 48 h of exposure. All values are results of three independent experiments (n = 3) with eight replicates and expressed as mean ± SD. *p* ≤ 0.05 (*), *p* ≤ 0.01 (**), and *p* ≤ 0.001 (***) corresponding to differences with respect to the control.

The cytotoxic effects of BEA, CTN, MON, and PAT were assessed in SH-SY5Y cells by the MTT assay after 24 h and 48 h of exposure (Figure 2), and the obtained medium inhibitory concentration (IC_50_) values are shown in Table 2.

The change in cell viability following exposure to BEA in SH-SY5Y cells is reported in Figure 2a. There was a significant increase in cell viability at concentrations ranging from 0.47 μM to 1.88 μM after 24 h (*p* ≤ 0.01) and 48 h (*p* ≤ 0.05) of exposure. At the highest concentrations tested, a marked decrease in the viability of these cells was observed, which was significant at both test times and for most of the concentrations studied. The minimum values reached after 24 h and 48 h of exposure were 18% (*p* ≤ 0.001) and 12% (*p* ≤ 0.001), respectively. In this case, the IC_50_ value was set at 12 μM and 3.25 μM after 24 h and 48 h of exposure, respectively (Table 2 and Figure 2a).

The results of exposure of SH-SY5Y cells to CTN are shown in Figure 2b, revealing several decreases in cell viability for 50 and 100 μM (45–50%) for both 24 h and 48 h of exposure. However, a slight increase in viability was observed for concentrations below 25 μM. The IC_50_ value was set at 80 μM and 50 μM after 24 h and 48 h of exposure, respectively (Table 2 and Figure 2b).

The exposure of SH-SY5Y cells to MON at the concentrations studied reached the IC50 value in very few conditions, as shown in Figure 2c. It can be observed that, after 24 h of exposure, there was a significant linear downward trend up to 51% viability, but not below this percentage. After 48 h of exposure, an increase in viability was observed that could be due to an adaptation or activation of the cellular defense mechanisms because of the exposure to this mycotoxin, a reason that can justify why the IC_50_ value was not reached after this exposure time (Table 2 and Figure 2c).

Figure 2d shows the cell viability values obtained after the use of PAT, revealing a clear decrease in cell viability from 0.19 μM to 3 μM that could be observed at both times tested, preceded by variable results at the lowest concentrations tested. At the highest concentration, viability decreased to a value of 17% (*p* ≤ 0.001) and 10% (*p* ≤ 0.001) after 24 h and 48 h of treatment, respectively. The IC_50_ value was set at 2.5 μM and 1.5 μM after 24 h and 48 h, respectively (Table 2 and Figure 2d).

### 2.3. Cytotoxicity Assay of the Binary Co-Exposure Treatment of BEA, CTN, MON, and PAT in the SH-SY5Y Cell Line

**Figure 3:**
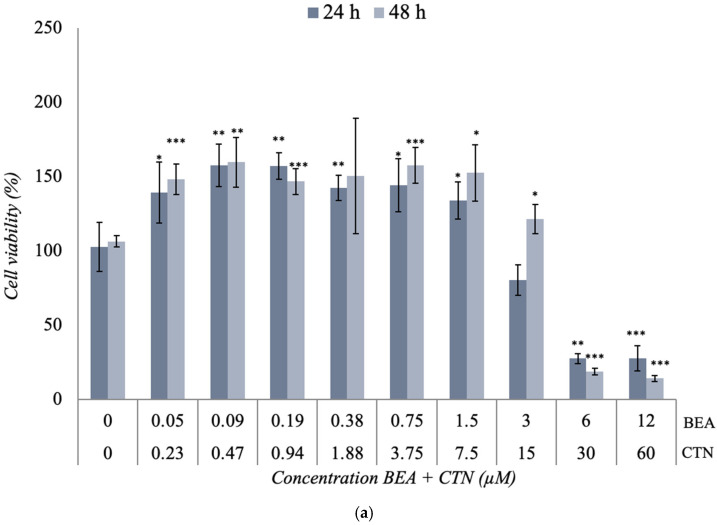

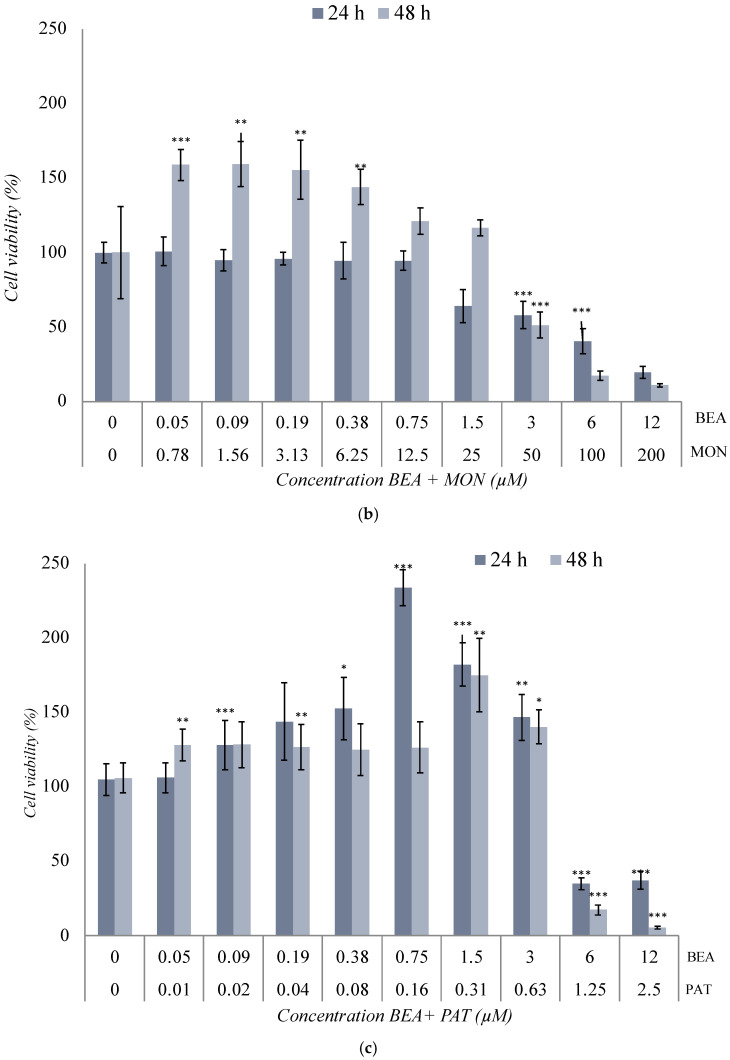

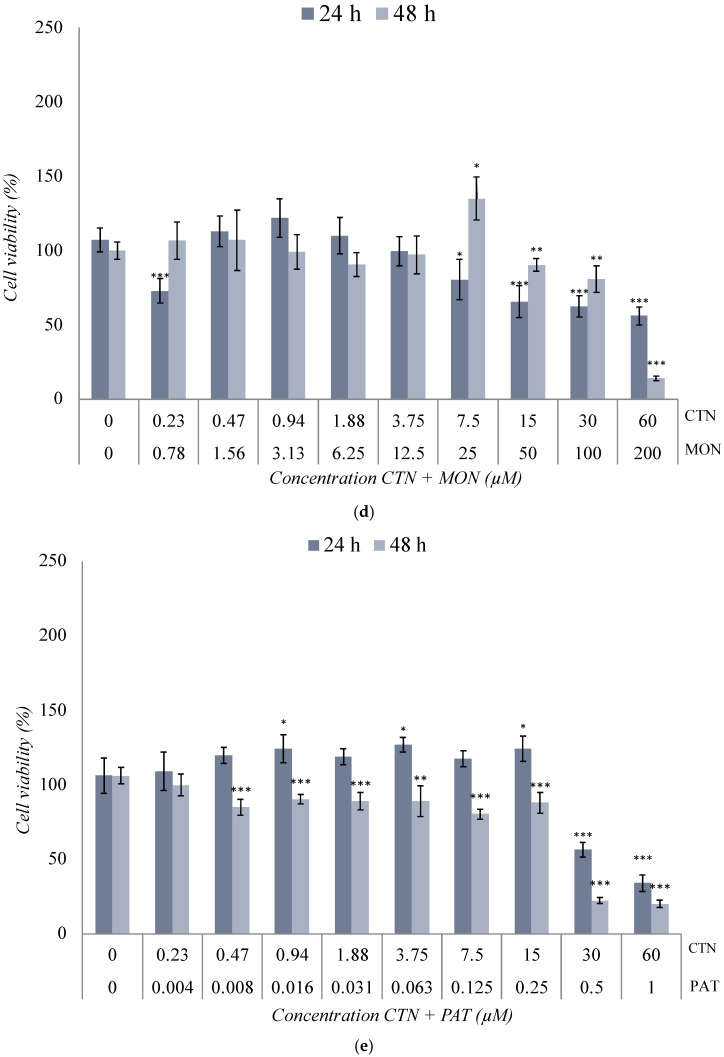

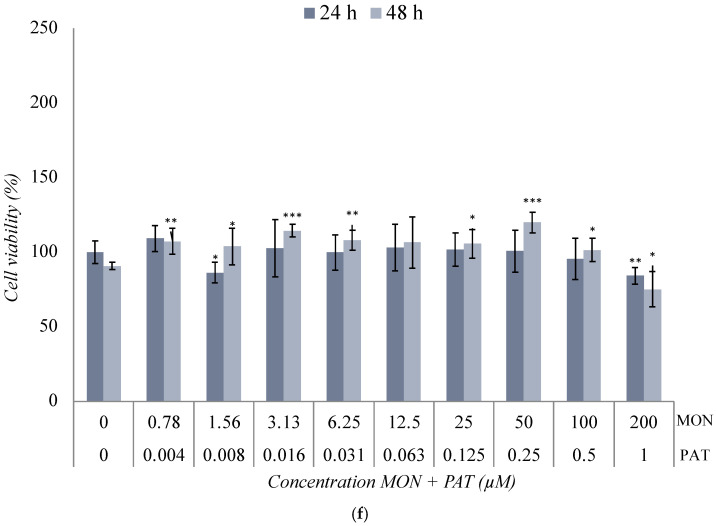
Cytotoxic effect of (**a**) BEA + CTN [1:5], (**b**) BEA + MON [3:50], (**c**) BEA + PAT [6:1.25], (**d**) CTN + MON [3:10], (**e**) CTN + PAT [60:1], and (**f**) MON + PAT [200:1] in SH-SY5Y monolayer cultures obtained by MTT assay after 24 h and 48 h of exposure. All values are results of three independent experiments (n = 3) with eight replicates and expressed as mean ± SD. *p* ≤ 0.05 (*), *p* ≤ 0.01 (**), and *p* ≤ 0.001 (***) corresponding to differences with respect to the control.

The combination of BEA + CTN [1:5] μM, presented in Figure 3a, showed a significant increase in viability at the first concentrations studied compared to the control. This increase was dropped down from [6 + 30] μM to a percentage of viability of 27% and 14% after 24 h and 48 h of exposure ([60 + 12] μM, (*p* ≤ 0.001)), respectively. In this case, the IC_50_ value was obtained at the concentrations of [4.5 + 22.5] μM and [4.8 + 24] μM after 24 h and 48 h, respectively (Table 2 and Figure 3a).

The combination of BEA + MON [3:50] μM in Figure 3b generated a significant decrease (*p* ≤ 0.001) in cell viability from [1.5 + 25] μM at the two exposure times used, reaching cell viability values of 19% and 10% after 24 h and 48 h of exposure, respectively (Figure 3b). However, at lower concentrations of exposure, cell viability was higher after 48 h compared to the results obtained after 24 h. This increase was up to 1.59-fold (*p* ≤ 0.001) higher than its control. In this case, the IC_50_ value was reached at [4.5 + 75] μM and [3 + 50] μM after 24 h and 48 h, respectively (Table 2).

The combination of BEA + PAT [6:1.25] μM in SH-SY5Y cells at the two times studied shows its results in Figure 3c. At the lowest concentrations of exposure, a somewhat linear behavior was observed. However, from [0.75 + 0.16], the viability increased significantly (*p* ≤ 0.001). Higher concentrations achieved a reduction in cell viability of 63% and 95% (*p* ≤ 0.01) after 24 h and 48 h of exposure, respectively. In this case, the IC_50_ value was set at [5.5 + 1.15] μM and [4.5 + 0.94] μM after 24 h and 48 h, respectively (Table 2 and Figure 3c).

The cytotoxic effect of the CTN + MON [3:10] μM combination is shown in Figure 3d. A slight decrease in cell viability was observed as the assayed concentration increased, which was significant from [15 + 50] μM for both exposure times. The inhibition of 50% of cell viability was only achieved at [45 + 150] μM after 48 h of exposure (Table 2 and Figure 3d).

The effect of the combination of CTN + PAT [60:1] μM in SH-SY5Y cells is shown in Figure 3e. This combination showed a higher toxicity than that obtained individually. The decrease in cell viability was marked at all concentrations tested after 48 h of exposure (*p* ≤ 0.001). In addition, a significant change (*p* ≤ 0.001) was observed from [30 + 0.5] μM at both 24 h and 48 h test times. In this case, the IC_50_ value was set at [45 + 0.75] μM and [22.5 + 0.38] μM after 24 h and 48 h of exposure, respectively (Table 2 and Figure 3e).

The cytotoxic effect of the binary combination of MON + PAT [200:1] μM is shown in Figure 3f. Cell viability was maintained without wide fluctuations for all concentrations tested and no IC_50_ was reached after any time of exposure (Table 2 and Figure 3f).

## 3. Discussion

Mycotoxin contamination of food and feed is a significant health concern today, and health risks associated with simultaneous exposure to multiple mycotoxins remain poorly understood and have not been extensively studied. As it is common for mycotoxins to appear together, monitoring them, both individually and in combination, is an essential task [9].

The evidence of the toxicity of these mycotoxins individually is shown in the literature and is summarized in Table 1. A large number of in vitro studies have been published, but there is a lack of research on the binary combination of BEA, CTN, MON, and PAT, although exposure to a combination of this type is very common. On the other hand, interest in mycotoxins that affect the neurological system has grown. This is why BEA, CTN, MON, and PAT appear to be strong candidates for investigating their single and combined effects and gaining a deeper understanding of their impact when exposed to SH-SY5Y cells.

Moreover, in this study, the cytotoxic effect of BEA, CTN, MON, and PAT and their binary combinations were measured through the MTT assay. This assay is effective in determining the concentrations that cause a 50% reduction in the percentage of cell viability and the maximum concentrations of compounds that are not toxic. For these reasons, it is an essential protocol in the evaluation of the sensitivity, in this case, of the cell line of interest to the mycotoxin under study. Moreover, it is a reference method, widely used in the literature, which allows for the comparison of the results obtained. The assays were conducted after 24 h and 48 h of exposure in accordance with protocols established in the reviewed literature, ensuring the consistency and comparability of the obtained results. These exposure times were chosen based on previous studies, allowing for a direct comparison of the cytotoxic effects under homogeneous experimental conditions.

All in all, this study constitutes a systematic screening approach to assess the effects of mycotoxins BEA, CTN, MON, and PAT on the SH-SY5Y cell line, providing an initial strategy to characterize the mycotoxins’ cytotoxicity using the MTT method. In this way, the results not only contribute to identifying the toxic effect of these substances, but also serve as a basis for further studies aimed at understanding the molecular mechanisms involved and how they contribute to the observed toxicity.

The cytotoxicity studies are key to protecting public health and establishing food safety regulations. The variability in cellular responses to mycotoxins highlights the need to consider which tissues are most vulnerable, such as the nervous system. This is crucial for preventing harm in vulnerable populations. These studies also help define exposure limits for mycotoxins, considering both dose and individual variability [72]. Additionally, knowledge about the most affected cells aids in designing preventive policies and regulating combined exposures to mycotoxins. Research improves methods for detecting and monitoring mycotoxins in food, using biomarkers of cellular damage to ensure food safety. This contributes to establishing more accurate and effective regulation [72,73].

### 3.1. Cytotoxicity Assays of Individual Treatment with BEA, CTN, MON, and PAT

SH-SY5Y cells exposed to BEA at concentrations ranging from 0 to 30 μM underwent a significant decrease in viability from a concentration of 1.88 μM onwards (Figure 2a). This significant decrease in SH-SY5Y cell viability upon exposure to BEA aligns with its known cytotoxic mechanisms [58]. As an ionophore, it increases the permeability of cell membranes to essential cations, leading to cellular dysfunction [4]. Results in other studies with different cell lines were close to those observed in our study. Specially, in the CHO-K1 cell line, the effect of BEA by obtaining the same IC_50_ value (12.08 μM) was corroborated [50]. For their part, Mallebrera et al. (2016) and Zouaoui et al. (2016) concluded that the decrease in cell viability was time-dependent and concentration-dependent for this same cell line [11,56]. Research by Juan-García et al. (2020) demonstrated a cell viability profile closely resembling that shown in Figure 2a, though it involved the HepG2 cell line [58]. The opposite was observed for activated genetic reported cells (RGA cell lines), as no cytotoxicity was observed in most cases and cell viability was maintained between 100% and 80% [57]. Furthermore, for Jurkat T cells [51] and pig small intestine epithelial cells (IPEC-J2) [59], cell viability results showed greater resistance to the effect of this mycotoxin, obtaining high [51] or not significant [59] cell viability percentages. These findings highlight a fairly heterogeneous cytotoxic profile of BEA. In fact, IPEC-J2, Jurkat-T, and RGA cell lines were among the most resistant to its effects, while the SH-SY5Y and HepG2 cell lines stood out for showing a more pronounced and very similar cytotoxicity profile. Finally, CHO-K1 distinguished itself as being the most sensitive cell line of those studied, according to the literature [50].

According to the available literature, the effect of the CTN mycotoxin exhibited significant variability depending on the cell line tested. The maximum concentration of CTN tested in this experimental study was 100 μM, achieving a percentage of cell viability close to 45% (Figure 2b). In line with the results obtained in the present work, other authors showed that the viability of SH-SY5Y cells after exposure to CTN decreased up to 95% after 24 h of exposure and for the maximum concentration studied (500 μM) [5], while, for a concentration of 50 μM, cell viability reached a value of 70% [24]. Tsai et al. (2023), for the same cell line, concluded that there was a 1.2-fold decrease in the value of the control at 50 μM after 72 h of exposure [26], and, at the higher concentration tested (80 μM), SH-SY5Y cell viability was 80% lower than the control [32]. In HepG2 cells, higher concentrations (300 μM) were used and high reductions in cell viability were achieved [48]. For the HEK293 cell line, CTN produced a reduction in viability in a concentration- and time-dependent manner, without reaching cell viability percentages below 60% after 24 h of exposure, but around 20% after 48 h of exposure [38]. For human colorectal carcinoma cells (HCT 116), concentrations of 150 μM reduced cell viability by 60% [61], while, for the FHS 74 Int cell line, no change in viability was observed [52], and, for Sertoli cells (TM4 Sertoli), cell viability was reduced by 25% to 65% [62]. As can be observed, the different cell lines and assays gave rise to variations in the results, which, although not very diverse, highlights the need for further studies on the behavior of CTN. Notably, it appears that the HCT 116 and TM4 Sertoli cell lines exhibit relatively higher resistance to CTN, whereas the SH-SY5Y cell line shows substantial reductions in viability, especially at higher concentrations of the mycotoxin, as corroborated by the present study. These findings are consistent with the well-established mechanisms of CTN toxicity, such as oxidative stress, mitochondrial dysfunction, and DNA damage, which have been implicated in its neurotoxic effects [26,31,32,33]. While the mechanisms involved have been widely studied in various in vitro models, our results further highlight the need to consider these pathways when assessing the impact of CTN on neuronal cells.

The effect of MON on human colon adenocarcinoma cells (Caco-2) revealed an inhibition in cell proliferation below 30% at a concentration of 100 μM [63]. However, for the same concentration, a 10% reduction in cell viability for male hamster lung cell line (V79) and CHO-K1 lines has been reported [63]. Similar results were found for human monocytic cell line (THP-1) at CTN 10 μM [64]; but, according to Smith et al. (2017), this same concentration caused only a 10% reduction in cell viability for human liver cell line (HepaRS) [65]. On the other hand, in the case of FHS 74 Int [52] and for pig small intestine epithelial cells (IPEC-J2) [59], exposure to MON did not affect cell viability in any case during the 72 h period. In our current study, using SH-SY5Y cells, the highest tested concentration of 200 μM MON led to a 50–60% retention of cell viability, while exposure to 100 μM resulted in a modest 10% reduction after 48 h of exposure (Figure 2c). These results are consistent with previous studies in other cell lines, where MON generally exhibited a comparatively lower cytotoxic effect relative to other mycotoxins explored in this study. Collectively, these findings suggest that MON tends to exert lower cytotoxicity compared to other mycotoxins, with the extent of cell viability reduction being concentration- and time-dependent. This supports the notion that MON may have a less potent cytotoxic profile, particularly at concentrations below 100 μM, across a range of cellular models.

In the case of PAT, cytotoxicity SH-SY5Y assays are shown in Figure 2d. The results agree with those found by other authors for V79 [67] and for the HEK293 cell line [38]. Percentages of cell viability 5-fold lower than their control were obtained after 24 h of exposure [67] and 10-fold lower after 24 h, 48 h, and 72 h of exposure [38], revealing a time-dependent trend. However, other authors stated that this decrease was concentration-dependent [37,49,68]. In the case of the transgenic mouse hepatocyte cell line (AML-12), PAT at concentrations of 4 μM reduced cell viability by 25% with respect to the control (after 36 h) [66]. For the glioblastoma cell line (DBTRG-05 MG), it was found that exposure to PAT generated a decrease of 95.8% for the maximum concentration tested (60 μM) after 24 h of exposure [41]. For the CHO-K1 cell line, the results indicated a marked decrease in cell viability, with IC_50_ values of 2.9 μM [11] and 0.69 μM [50]. Finally, Pal et al. (2022) concluded that only PAT concentrations of 1–5 μM significantly reduce cell viability, but not concentrations lower than these [69]. After analyzing all the results, it can be stated that PAT is a mycotoxin with a high cytotoxic capacity and that, at low concentrations, it shows significant effects on most of the cell lines studied. In relation to this, the SH-SY5Y cell line and CHO-K1 cell line could be considered as the most sensitive to the effect of this toxin. On the other hand, and according to what has been mentioned, the DBTRG-05MG cell line would be the most resistant of those involved in the study, although it is true that all of them see their viability reduced after exposure to concentrations that turn out to be lower than those used in tests of other mycotoxins.

After assessing the cytotoxicity of the mycotoxins of interest in isolation, a rank order of toxicity can be established, with patulin (PAT) at the forefront due to its pronounced toxic effects across the majority of cell lines examined. PAT consistently demonstrated significant reductions in cell viability, indicating its high cytotoxic potential. Following PAT, BEA and CTN exhibit moderate toxicity, although the data across the different cell lines showed some degree of heterogeneity, suggesting variability in the cytotoxic response. These mycotoxins generally produced notable toxicity but did not reach the levels observed with PAT, which may be attributed to differences in their mechanisms of action and cellular targets. Lastly, MON emerged as the least toxic mycotoxin in this study. The decrease in cell viability was comparatively minimal, even at higher concentrations, highlighting its relatively low cytotoxic potential. The consistently low toxicity observed across various cell lines further corroborates MON’s position as the least cytotoxic mycotoxin among those tested. This indicates that MON may have a significantly lower impact on cellular integrity and function, at least within the tested concentration range, when compared to the other mycotoxins evaluated in this study.

### 3.2. Cytotoxicity Assays of the Binary Co-Exposure Treatment of BEA, CTN, MON, and PAT

Existing research on mycotoxin combinations is limited, even though the realistic nature of simultaneous exposure to mycotoxins is known. This is due to the complexity of their interactions, since they do not follow a predictable pattern; mycotoxin diversity presents limitations in available analytical methods [71]. On the other hand, the lack of specific regulations around the ceilings in combination has made research into them not a priority. Thus, the comparison of the results obtained (Figure 3; Table 2) with other findings reported in the literature is not possible for most of the binary combinations presented.

The literature on the effects of BEA + CTN (Figure 3a) is scarce; in fact, the lack of in vitro experiments studying this particular combination does not allow for comparison. Remarkably, given that BEA alone exhibited greater toxicity than CTN, it can be argued that the latter increases its toxicity in combination with BEA, obtaining significantly low percentages of cell viability at the highest concentrations of exposure. Interestingly, exposure to BEA + PAT (Figure 3c) generally increased cell viability compared to exposure to the mycotoxins individually (Figure 2a,d). However, as corroborated by other authors for the same combination of mycotoxins [11], cell viability was found to be decreased at the highest exposure concentrations. This could indicate the existence of a protective interaction at the lower concentrations used for the two mycotoxins, since similar cytotoxicity profiles were obtained. These two mycotoxins showed a weakening of their cytotoxic power at lower concentrations of exposure compared to their control. On the other hand, this event is reversed by a potentiation of both toxins at the highest exposure concentrations (Figure 3a,c).

As for the combination of CTN + PAT (Figure 3e), the literature results (Table 1) showed a reduction in cell viability in the bovine macrophage cell line (BOMACs) of 50% for [52. 72 + 0.32] μM after 48 h of exposure [60]. The same percentage of reduction in cell viability was given for [45 + 0.75] μM, following exposure of the SH-SY5Y cell line to this combination of mycotoxins. In addition, the binary combination showed lower cell viability than the mycotoxins individually, especially after 48 h. In this direction, mycotoxins in combination manage to strengthen their effects, giving rise to a greater cytotoxic power.

Lastly, the results of mycotoxins’ combination with MON were diverse. On the one hand, the combination of MON + PAT did not generate highly significant variations with respect to the control (Figure 3f). As for BEA + MON (Figure 3b), the general trend corresponded to a significant decrease in cell viability. However, the combination of CTN + MON (Figure 3d) showed similar results to those obtained after the individual treatment with MON. From these findings, it can be deduced that the low toxicity of MON played a fundamental role in the results obtained for the combinations of MON + PAT (Figure 3f) and CTN + MON (Figure 3d), overshadowing the cytotoxic effect shown by PAT and CTN individually (Figure 2). The toxicity of MON, individually, has been studied on multiple occasions and, in all of them, it has generated small decreases in the percentage of cell viability, regardless of the concentration used [52,59,63,64,65]. Furthermore, it is remarkably difficulty to obtain IC_50_ values when combining different mycotoxins with MON (Table 2), as seen in the results of individual exposure to MON (Table 2). The lack of IC_50_ values for MON combinations could be due to the inherently low toxicity of it, which, even at high concentrations, fails to produce significant reductions in cell viability. Moreover, MON may act as a modulator of the toxicity of other mycotoxins, reducing their cytotoxic effect through a potential antagonistic interaction. Thus, it can be stated that CTN and PAT had their cytotoxic effect diminished after their combination with MON (Figure 3d,f). However, the effect of the BEA + MON combination was completely opposite. In this case, it can be said that BEA is the mycotoxin that exerts the primary effect, due to the large reduction obtained in the percentage of cell viability. In addition, these mycotoxins show the potentiation of their effect on SH-SY5Y cells in combination compared to the results with those obtained after exposure individually. Similar results were found for the binary combinations of α-ZEA and β-ZEL with BEA, since the high toxicity of BEA played a fundamental role after exposure of the SH-SY5Y cell line to the two binary combinations [21]. BEA and PAT are two mycotoxins that share common toxicity mechanisms, including oxidative stress and mitochondrial dysfunction. These shared mechanisms may contribute to the enhanced cytotoxicity observed when the mycotoxins are combined, as an exacerbation of these processes could occur. That said, it can be stated that this combination stands out as one of the most toxic according to the findings of this study.

The combinations of BEA + MON and CTN + PAT resulted in a greater reduction in cell viability than anticipated, suggesting that their co-exposure may influence cytotoxic outcomes beyond an additive effect. In the case of BEA + MON, the pronounced decrease in cell viability indicates that BEA exerts the primary toxic effect, while MON, despite its typically low cytotoxicity, may modulate this response through mechanisms such as altered cellular uptake or metabolic interactions. Similarly, CTN + PAT exposure led to a more pronounced cytotoxic effect than individual treatments, potentially due to disruptions in cellular homeostasis involving oxidative stress, mitochondrial dysfunction, or interference with key survival pathways. On the contrary, the combination of BEA + PAT generates lower toxicity than when the mycotoxins are tested individually. This phenomenon might be due to the modulation of specific cellular pathways that mitigate the toxic effects, possibly through mechanisms such as antioxidant activity, altered apoptosis signaling, or the inhibition of certain pro-inflammatory responses. Notably, this suggests that the effects of combined exposure are not simply a sum of their individual toxicities. Instead, the combined presence of these mycotoxins may result in complex interactions that could either mitigate or amplify their individual effects depending on the concentration. These findings underscore the complexity of mycotoxin interactions and highlight the need for further mechanistic studies to elucidate the underlying cellular and molecular processes governing their combined toxicity. Moreover, although tertiary (three mycotoxins) and even quaternary (four or more mycotoxins) combinations have been detected, they appear less frequently. Advances in analytical methods have made it possible to identify multiple mycotoxins in certain matrices and regions, but the literature generally supports the fact that binary combinations remain the most prevalent. As a result, this information and the results obtained are particularly relevant for public health. Even though exposure to more than two mycotoxins can potentially enhance toxic effects through synergistic interactions, the higher incidence of binary combinations means that they are the primary focus of monitoring and regulatory strategies.

## 4. Conclusions

Exposure to mycotoxins poses a major challenge to global food safety, endangering human and animal health. PAT treatment exhibited the highest cytotoxicity even at low concentrations, while BEA and CTN showed intermediate toxicity, and MON had the lowest toxicity. Among binary combinations, BEA + MON and CTN + PAT were highly toxic, whereas MON + PAT and CTN + MON showed low cytotoxicity. Interestingly, BEA + CTN and BEA + PAT increased cell viability at low concentrations, suggesting a protective interaction. These findings highlight the need for further research on mycotoxin effects and interactions. Additionally, they provide valuable insights for improving food safety, establishing permissible limits, and strengthening regulatory frameworks to reduce mycotoxin exposure and protect public health.

## 5. Materials and Methods

### 5.1. Chemical Reagents

The chemical reagents’ grade and cell culture components used were Dulbecco’s Modified Eagle’s Medium-F12 (DMEM/F-12), phosphate buffer saline (PBS), and fetal bovine serum (FBS) purchased from Thermo Fisher, GibcoTM (Paisley, UK). MTT for cytotoxicity assays, penicillin, streptomycin, and Trypsin/EDTA solutions were purchased from Sigma-Aldrich (St. Louis, MO, USA). Dimethyl sulfoxide (DMSO) and methanol (MeOH) were obtained from Fisher Scientific Co, Fisher BioReagentsTM (Geel, Belgium). Mycotoxins BEA, CTN, MON, and PAT were acquired from Sigma Aldrich (St. Louis, MO, USA). Stock solutions of mycotoxins BEA, CTN, and MON were prepared in DMSO and maintained at −20 °C in the dark. Moreover, the stock solution of PAT was prepared in MeOH and maintained at −20 °C in the dark. The final mycotoxin solvent concentration in the medium was ≤1% (*v*/*v*) as pre-established [74]. All other reagents were taken from the laboratory grade.

### 5.2. Cell Culture

Undifferentiated human neuroblastoma cell line SH-SY5Y was purchased from the American Type Culture Collection (ATCC, Manassas, VA, USA) and seeded using DMEM/F12 culture medium supplemented with 10% FBS and 1% of the mixture of penicillin (100 U/mL) and streptomycin (100 mg/mL). Cells were left to reach confluence of 80–90% and, subsequently, were treated with trypsin once or twice a week while being suspended in complete medium in a 1:3 split ratio with a maximum of 20 cell passages. Cell cultures were incubated at 37 °C and 5% CO_2_ atmosphere.

### 5.3. Mycotoxin Exposure

Mycotoxin stock solutions (BEA, CTN, MON, and PAT) were prepared in DMSO and MeOH and kept at −20 °C preserved from light. BEA, CTN, and MON purchased powders were resuspended to a final concentration of 7000 µM, 10,000 µM, and 20,000 µM, respectively, with DMSO, prepared from those of the initial tested concentration (Table 3). On the other hand, PAT was resuspended with MeOH to a final concentration of 1000 µM with MeOH also prepared from the initial tested concentration. Afterward, intermediate solutions were prepared from the initial solutions to achieve the studied concentrations of the forementioned mycotoxins.

Cells were cultured in 96-well plates at 2 × 10^4^ cells/well until reaching a confluence of 80–90% in the well before the exposure to mycotoxins for 24 h and 48 h. Mycotoxins BEA, CTN, MON, and PAT were tested individually at concentration ranges of 0.12–30 μM, 0.39–100 μM, 0.78–200 μM, and 0.01–3 μM, respectively (Table 3). A total of eight serial dilutions (1:2 dilutions) were tested from the initial to the final concentration.

The binary combinations of mycotoxins used in the treatment of SH-SY5Y were BEA + CTN; BEA + MON; BEA + PAT; CTN + MON; CTN + PAT; and MON + PAT at the concentration ranges 0.28–72 μM, 0.83–212 μM, 0.06–14.5 μM, 1.01–260 μM, 0.234–61 μM, and 0.784–201 μM, respectively. In the same way, eight dilutions of each mycotoxin concentration were tested at two exposure times (24 h and 48 h) (Table 3). The concentration chosen for each mixture was based on the range that ensured that viability did not reach a value below IC_50_ (Table 2) after its individual assay. Selecting concentrations below the individual IC_50_ ensures the avoidance of excessive reductions in cell viability, which could compromise the integrity of toxicological responses and lead to non-reproducible or biologically irrelevant data. This approach is critical for delineating interactions between mycotoxins given the similarity to realistic conditions, providing insights into the combined toxicological effects of mycotoxins under circumstances that are representative of actual exposure events. Additionally, maintaining cell viability above the IC_50_ threshold is consistent with standard toxicological practices [32,43,75]. This approach is carried out to avoid non-specific effects and ensure the biological relevance of the results. The dilution ratios for the binary combinations were 1:5 for BEA + CTN, 3:50 for BEA + MON, 6:1,25 for BEA + PAT, 3:10 for CTN + MON, 60:1 for CTN + PAT, and 200:1 for MON + PAT. The dilution ratios of the concentrations for the binary combinations were, as explained above, based on the results of the individual treatment. The solvent control was DMSO or MeOH depending on the mycotoxin or combination of mycotoxins, as described in Section 2.1. In all cases, the percentage of this solvent remained ≤1%.

### 5.4. MTT Assay

MTT is based on a colorimetric reaction characterized by converting the MTT reagent, in its yellow oxidized form, into insoluble formazan crystals, its reduced and purple form. The procedure described by Juan-García et al. (2015) with some modifications was used for the evaluation of the cytotoxicity of the mycotoxins BEA, CTN, MON, and PAT and their binary combinations [76]. The concentration of cells/well seeded in 96-well plates was 2 × 10^4^ cells/well. Cells were left to incubate until reaching a confluence of 80–90% in the well and, after the incubation time, they were treated with mycotoxins BEA, CTN, MON, and PAT individually and with their binary combinations. During the mycotoxin exposure time (24 h and 48 h), neither the medium nor the mycotoxins were replenished according to the MTT protocol. After the 24 h and 48 h of exposure, the medium was removed from the wells, 200 μL of fresh medium/well and 50 μL of MTT dissolved in PBS at a concentration of 5 mg/mL were added to each well. The plates were then incubated for 4 h at 37 °C. Finally, the content of the wells was removed and 200 μL of DMSO and 25 μL of Glycine–Sorensen solution were added to solubilize the formazan crystals. Absorbance was read at 570 nm using Multiskan EX (Thermo Scientific, Waltham, MA, USA) and Ascent Software Version 2.6 software. All experiments were tested with eight replicates each time and cell viability was expressed in percent relative to control cell. From concentration–effect curves, the IC_50_ values were calculated.

### 5.5. Statical Analysis

Data statistical analysis was performed using SPSS version 13 (SPSS, Chicago, IL, USA). Data were expressed as mean ± SD of three independent experiments, and IC_50_ values were estimated using linear interpolation. Statistical analysis was conducted using Student’s *t*-test for paired samples, and a one-sample *t*-test was used when comparing the dataset to the control group. Data points deviating from the expected range were excluded to maintain data integrity and reliability. A *p*-value of ≤0.05 was considered statistically significant.

## Figures and Tables

**Table 2:** Medium inhibitory concentration (IC_50_ ± SD) of beauvericin (BEA), citrinin (CTN), moniliformin (MON), and patulin (PAT) and their binary combination for SH-SY5Y cells after 24 h and 48 h of exposure, determined by the MTT assay. All values are results of three independent experiments (n = 3) with eight replicates and expressed as mean ± SD.

Mycotoxin	Exposure Time 24 h IC_50_ (Mean ± SD) (μM)	Exposure Time 48 h IC_50_ (Mean ± SD) (μM)
BEA	(12 ± 8)	(3.25 ± 6.36)
CTN	(80 ± 2)	(50 ± 4)
MON	(200 ± 6)	N/A
PAT	(2.5 ± 1)	(1.5 ± 4.5)
BEA + CTN	[4.5 + 22.5] ± 3.4	[4.8 + 24] ± 2.2
BEA + MON	[4.5 + 75] ± 8.5	[3 + 50] ± 3
BEA + PAT	[5.5 + 1.15] ± 4.02	[4.5 + 0.94] ± 3.27
CTN + MON	N/A	[45 + 15] ± 2
CTN + PAT	[45 + 0.75] ± 4.80	[22.5 + 0.38] ± 1.98
MON + PAT	N/A	N/A

N/A: not achieved.

**Table 3:** Concentration range (µM) of mycotoxins studied individually and in binary combinations.

Mycotoxin (Dilution Ratios)	Concentration Range (μM)
BEA	0.12–30
CTN	0.39–100
MON	0.78–200
PAT	0.01–3
BEA + CTN (1:5)	0.28–72
BEA + MON (3:50)	0.83–212
BEA + PAT (6:1.25)	0.06–14.5
CTN + MON (3:10)	1.01–260
CTN + PAT (60:1)	0.234–61
MON + PAT (200:1)	0.784–201

## Data Availability

The original contributions presented in this study are included in the article. Further inquiries can be directed to the corresponding author.
